# Same Difference? Low and High Glucosinolate *Brassica rapa* Varieties Show Similar Responses Upon Feeding by Two Specialist Root Herbivores

**DOI:** 10.3389/fpls.2019.01451

**Published:** 2019-11-13

**Authors:** Rebekka Sontowski, Nicola J. Gorringe, Stefanie Pencs, Andreas Schedl, Axel J. Touw, Nicole M. van Dam

**Affiliations:** ^1^Molecular Interaction Ecology, German Centre for Integrative Biodiversity Research Halle-Jena-Leipzig, Leipzig, Germany; ^2^Institute for Biodiversity, Friedrich Schiller University, Jena, Germany; ^3^School of Biosciences, Cardiff University, Cardiff, Wales, United Kingdom

**Keywords:** plant–insect interactions, belowground herbivory, glucosinolate transporters, herbivore-induced plant responses, insects

## Abstract

Glucosinolates (GSLs) evolved in Brassicaceae as chemical defenses against herbivores. The GSL content in plants is affected by both abiotic and biotic factors, but also depends on the genetic background of the plant. Since the bitter taste of GSLs can be unfavorable for both livestock and human consumption, several plant varieties with low GSL seed or leaf content have been bred. Due to their lower GSL levels, such varieties can be more susceptible to herbivore pests. However, low GSL varieties may quickly increase GSL levels upon herbivore feeding by activating GSL biosynthesis, hydrolysis, or transporter genes. To analyze differences in herbivore-induced GSL responses in relation to constitutive GSL levels, we selected four *Brassica rapa* varieties, containing either low or high root GSL levels. Plants were infested either with *Delia radicum* or *Delia floralis* larvae. The larvae of both root flies are specialists on *Brassica* plants. Root samples were collected after 3, 5, and 7 days. We compared the effect of root herbivore damage on the expression of GSL biosynthesis (*CYP79A1*, *CYP83B2*), transporter (*GTR1A2*, *GTR2A2*), and GSL hydrolysis genes (*PEN2, TGG2*) in roots of low and high GSL varieties in conjugation with their GSL levels. We found that roots of high GSL varieties contained higher levels of aliphatic, indole, and benzyl GSLs than low GSL varieties. Infestation with *D. radicum* larvae led to upregulation of indole GSL synthesis genes in low and high GSL varieties. High GSL varieties showed no or later responses than low varieties to *D. floralis* herbivory. Low GSL varieties additionally upregulated the GSL transporter gene expression. Low GSL varieties did not show a stronger herbivore-induced response than high GSL varieties, which indicates that there is no trade-off between constitutive and induced GSLs.

## Introduction

The Brassicaceae family contains many economically important plant crops with more than 170 million tons of cultivated vegetables and oilseeds produced worldwide each year (Fao, 2017). One intensively cultivated species of this family is *Brassica rapa* L., which has a long history of domestication in Europe (Warwick, 2011)*.* *B. rapa* is cultivated for livestock and human consumption and includes many well-known varieties, such as turnip, pak choi, Chinese cabbage, and field-mustard. Humans and livestock as well as insects and microbes exploit these plants as food source. To defend themselves against herbivores, plants in the Brassicaceae produce glucosinolates (GSLs), a group of plant secondary metabolites (Jeschke and Burow, 2018). These compounds are located in all parts of the plants including in the roots. More than 130 GSLs have been identified to date (Agerbirk and Olsen, 2012). GSLs are generally categorized as aliphatic, indole, or benzyl (also referred to as aromatic) GSLs, depending on their amino acid precursors (Fahey et al., 2001). When plant tissue is damaged, myrosinases hydrolyze GSLs to form isothiocyanates, nitriles, thiocyanates, and other biologically active compounds (Bones and Rossiter, 2006). Myrosinases are broad-spectrum ß-thioglucosidases with a high affinity for a wide range of GSLs. Up- and downregulation of myrosinase transcripts upon aboveground herbivory has been described for *Brassica napus* [reviewed in Textor and Gershenzon (2009)]. The atypical ß-thioglucosidase PEN2 specifically hydrolyzes indole GSLs after which unstable isothiocyanates are formed (Bednarek et al., 2009). The latter spontaneously form carbinols, or are conjugated to glutathione and then converted to amines and structurally related indole acids, depending on the presence of modifying enzymes in the plant (Wittstock and Burow, 2010). Many of these hydrolysis products have defensive properties against a range of arthropods, microbes, and nematodes (Potter et al., 1998; Kim and Jander, 2007; Clay et al., 2009). Aliphatic GSLs and their breakdown products negatively affect chewing insects and microbes (Li et al., 2000; Arany et al., 2008). Indole GSLs play a role in pathogen defense and provide resistance to phloem-feeding insects and microbes (Agerbirk et al., 2009), whereas benzyl GSLs provide resistance to nematodes (Potter et al., 1999; Kabouw et al., 2010). Although GSLs are produced constitutively, their biosynthesis and that of ß-thioglucosidases can be induced during interactions with pest and pathogens as well (Brader et al., 2006; van Geem et al., 2015; Zhang et al., 2016). Upon herbivory, GSL levels can increase both locally and systemically (Textor and Gershenzon, 2009). Local GSL accumulation can be achieved by local biosynthesis or transport from organ to organ by specific transporter proteins (Nour-Eldin and Halkier, 2009). However, induced GSLs can in turn be hydrolyzed and released upon herbivory based on the higher anti-herbivore function of the hydrolysis products (Li et al., 2000). This process may result in lower or absent GSL accumulation in local tissues.

Although many studies have investigated the interactions between Brassicaceae and aboveground insects, interactions with belowground insects are less well studied. Two common specialists on *Brassica* plants are the cabbage root fly (*Delia radicum* L.) and the turnip root fly (*Delia floralis* Fall. Diptera: Anthomyiidae). After hatching at the interface between the lower part of the stem and the roots, the larvae move into the soil where they mine into the taproot (Dosdall et al., 1994). Because of the extensive damage the larvae can cause to the root system, both species are considered important pests in agriculture (Finch and Collier, 2000; Bjorkman et al., 2010). Plants respond to *D. radicum* and *D. floralis* attacks with local and systemic induction of GSLs in roots and leaves (Birch et al., 1992; Pierre et al., 2011; Tsunoda et al., 2018). This GSL accumulation can be caused by both an increase in GSL biosynthesis, by GSL transport from systemic parts such as other root parts, leaves or the stem, or a combination of both (Andersen et al., 2013). However, it is not yet known how the activation of GSL biosynthesis and transport mechanisms are connected.

All Brassicaceae produce GSLs, but GSL concentration and composition can greatly vary among varieties depending on their genetic background (Giamoustaris and Mithen, 1995;Wurst et al., 2006; del Carmen Martínez-Ballesta et al., 2013). Breeders have selected for varieties with high or low GSL levels, depending on consumers preference and the presence of specific GSLs. For example, consumers prefer a strong and bitter taste in some crops, such as mustard, wasabi, or horseradish. Other GSLs, such as glucoraphanin, are selected for because their breakdown products enhancement of human health (Fahey et al., 1997). In contrast, in oil seed rape breeders have selected for low levels of the 2OH-glucosinolate progoitrin, because of its hazardous effect on human health (Hopkins et al., 2009). However, these low GSL varieties may be more susceptible to herbivory due to low levels of constitutive GSLs (Glen et al., 1990; Beekwilder et al., 2008; Baaij et al., 2018). To compensate, varieties with low constitutive GSL concentrations may respond more strongly to herbivory than varieties with high constitutive GSL concentrations. Such trade-offs between constitutive and inducible defenses to aboveground herbivores have been experimentally assessed in *Arabidopsis thaliana* L. accessions differing in shoot GSL levels (Rasmann et al., 2015). To address whether trade-offs may also occur for belowground GSLs, we compared induced responses to the belowground feeding specialists *D. radicum* and *D. floralis* larvae in low and high GSL *B. rapa* varieties. We postulated that low GSL varieties respond faster and more strongly to root herbivory than high GSL varieties. Moreover, we hypothesized that the response to both root fly species, which have comparable feeding strategies, would be similar. We tested these hypotheses by measuring the temporal expression dynamics of genes involved in GSL biosynthesis, transport, and hydrolysis. Furthermore, we measured root GSL profiles before and after herbivory. This allowed us to compare gene expression patterns to changes in the level of different GSL classes in roots, and compare these between high and low GSL varieties.

## Materials and Methods

### Insect Cultures

*D. radicum* and *D. floralis* larvae originated from our lab culture; a starting culture of *D. radicum* was kindly provided by Anne-Marie Cortesero, University of Rennes, France in 2014. Pupae for the *D. floralis* starting culture were provided by Maria Björkman, Bioforsk – Norwegian Institute for Agricultural and Environmental Research, Norway in 2015. Both species were reared for more than two years in our lab under the same conditions in a controlled environment cabinet (Percival Scientific, Perry, Iowa, USA) at a constant temperature of 20°C ± 2°C, 85% ± 8% relative humidity and under a 16/8 h day/night light cycle with a light intensity of 69 ± 18 µmol s^−1^ m^−2^. Larvae were reared in small plastic containers (10 × 10 × 6 cm) filled with 2 cm autoclaved sand on commercially bought kohlrabi (*Brassica oleracea*). Sand was kept moist and fresh kohlrabi pieces were provided every other day. Old pieces of uneaten kohlrabi which were left by the larvae, were removed. After about 24 days, the larvae crawl into the sand to pupae. Three days later, the pupae were removed from the sand by flooding the sand with tap water and decanted the water including swimming pupae with a small sieve. After about 3 weeks, adult flies emerged and transferred to a net cage (40 × 40 × 90 cm), where they were mass reared on a honey–water mix on cotton and a milk powder (Peter Knoll GmbH & Co KGaA, Elmshorn, Germany) yeast flakes (Sanotact GmbH, Muenster, Germany) mixture (1:1) offered in an open petri dish (8 cm diameter). The fly diet was exchanged for fresh diet twice a week. A box (10 × 10× 6 cm) filled with 2 cm moistened sand and a piece of kohlrabi was placed in the cage for the flies to oviposit on. The oviposition box was exchanged for a new box twice a week. Boxes that were removed from the cage, were closed with clear plastic lid, with 5 × 5 cm square in the middle, covered with gauge for aeration. The boxes with the eggs were placed back into the rearing cabinet. Larvae used for the experiment were reared on a semi-artificial diet containing 4% yeast extract (Carl Roth GmbH & Co KG, Karlsruhe, Germany), 4% lactose (Carl Roth GmbH & Co KG, Karlsruhe, Germany), 2% agar–agar plant (Carl Roth GmbH & Co KG, Karlsruhe, Germany), and 4% freeze-dried kohlrabi (commercially bought) in distilled water. This was done to minimize food adaptation.

### Selection of High and Low GSL Varieties

#### Experimental Set-Up—Plant Growth and Selection of Varieties

To select *B. rapa* varieties with low and high root GSL levels, we purchased seeds from nine randomly selected *B. rapa* varieties from the seedbank IPK Gatersleben, Germany. In addition we used one wild variety propagated in-house which originated from a natural population in Maarssen, The Netherlands (Baaij et al., 2018; **Table S1**). Seeds were germinated in vermiculite (1–2 mm, duengerexperte.de, Attenzell, Germany) at 20°C ± 2°C and 85% ± 8% relative humidity in a controlled environment cabinet (Percival Scientific, Perry, Iowa, USA). After 17 days, 10 seedlings from each variety (in total 100 seedlings) were transferred to a greenhouse, repotted in a sand-B pot clay medium mix (1:1, commercially bought by Gerhard Rösl GmbH, Jesewitz and Baywa AG, Laussig, Germany), and fertilized with Osmotcote^®^ pro 3-4M (Hermann Meyer KG, Rellingen, Germany). Plants were grown in the greenhouse of the Botanical Gardens, Leipzig, Germany, at 24°C ± 3°C, a relative humidity from 6% to 52%, under artificial lights (metal halogen vapor lamp Master HPI-T plus 400W, Phillips, Hamburg, Germany) set to maintain day length of 16 h. The plants were watered when needed but at least twice a week. Temperature and relative humidity were recorded every 12 min. After 6 weeks, the third fully expanded leaf and the roots were harvested from plants at a similar growth stage which had developed three pairs of leaves (BBCH code 13). All leaves were harvested by cutting them at the base of the petiole with a sharp razor, after which they were flash-frozen in liquid nitrogen. Roots were removed from the pots, rinsed with cold tap water in a bucket to remove soil particles from the fine roots, dried with tissue to remove excess water, and flash-frozen in liquid nitrogen as a whole. The samples were kept at −80°C until they were freeze-dried to constant weight. The dried leaf and root samples were ground to fine powder using a ball mill (Retsch MM 400, Retsch GmbH, Haan, Germany). An aliquot of the dried and ground root and leaf samples was extracted and analyzed for GSLs as below. In total 10 replicates of roots and leaves per variety were extracted and analyzed. Based on the total GSL concentrations in roots (**Figure S1**), we selected two *B. rapa* varieties with a low (variety A and B) and two with a high total GSL concentration (variety D and E) for the main experiment.

#### GSL Extraction and Quantification

The GSLs were extracted from 100 ± 5 mg of ground leaf or root samples according to Grosser and van Dam (2017). Briefly, GSLs were extracted in 70% methanol and the supernatant was transferred to an ion-exchange column (DEAE-Sephadex, Merck KGaA, Darmstadt, Germany). GSLs were desulfated with aryl sulfatase (type H-1 from *Helix pomatia*, Merck, Darmstadt, Germany). Desulfated GSLs were analyzed on high-performance liquid chromatography (HPLC) system equipped with a photodiode array detector (PDA; Ultimate 3000 series system DAD-3000(RS), Thermo Fisher Scientific, Waltham, MA, USA). HPLC-grade solvents were used throughout the analysis. Injection volume was set to 10 µl. Desulfated GSLs were separated on a reverse phase C_18_ column (Acclaim™ 300 C18 column, 4.6 × 150 mm, 300 Å, 5 µm, Thermo Fisher Scientific, Waltham, MA, USA) plus a C_18_ precolumn (10 × 4.6 mm, 5 µm particle size) using an acetonitrile-water gradient (Grosser and van Dam, 2017) at a flow of 0.75 ml/min and a column temperature of 40°C. Desulfated GSLs were identified based on retention time and UV spectra compared to commercial available reference standards (Phytoplan Diehm & Neuberger GmbH, Heidelberg, Germany, summarized in **Table S2**). GSLs were quantified at 229 nm using sinigrin as an external standard and response factors as in Grosser and van Dam (2017). Data were processed using Thermo Sientific Chromeleon Chromatography Data System software [version 7.2 SR5 MUa (9624) Thermo Fisher Scientific, Waltham, MA, USA].

### Root Fly Induced GSL Responses in High and Low GSL Varieties

#### Experimental Set-Up

In the main experiment, seeds of the four selected *B. rapa* varieties were germinated and grown as described earlier. The roots of 7- to 8-week-old plants were infested with five, second-instar larvae of *D. radicum* or *D. floralis.* Plants were randomly assigned to control, *D. radicum*, or *D. floralis* treatments. To infest a plant, the soil was carefully removed from around the root–shoot interface, after which five individual *D. radicum* or *D. floralis* larvae were added directly to the root system using a soft paint brush. Control plants were not infested. Harvesting took place 3, 5, and 7 days after the larvae were placed on the plants. To prevent that seasonal effects would affect our results, plants were grown in four batches between December 2017 and May 2018. Each batch included at least 18 plants per variety, including 2 plants per time point and treatment group. For this experiment only roots were harvested using the same procedure as during the selection of high and low GSL varieties. All *D. radicum* and *D. floralis* infested roots showed a visible feeding damage on the outside of the roots. After rinsing, the roots were flash-frozen in liquid nitrogen, freeze-dried, and ground. All together, we harvested eight replicates per treatment and variety at each time point after the start of herbivory (in total 288 plants). The samples of the two plants harvested per treatment at each time point in one and the same batch were pooled and used for all following extractions (in total 144 samples). This resulted in four replicates per treatment, time point, and variety.

#### Gene Expression Analysis

To study the molecular mechanisms that underlie the accumulation of GSLs in high and low GSL varieties in response to specialist root herbivores, we analyzed the expression of selected genes involved in the GSL biosynthesis, transport, and hydrolysis using a quantitative PCR (qPCR) procedure. We selected *CYP83A1* as marker gene for the aliphatic GSL biosynthesis and *CYP79B2* for the indole GSL biosynthesis (Mathur et al., 2013). We selected *GTR1A2* and *GTR2A2* expression as markers for the GSL transport activity. Both transporters are involved in the transport of aliphatic and indole GSLs (Jørgensen et al., 2017). We selected *TGG2* and *PEN2* as marker genes for GSL hydrolysis. *TGG2* encodes for myrosinase, a general GSL hydrolase (Xue et al., 1995). *PEN2* is specific for indole GSL hydrolysis (Wittstock and Burow, 2010). Using Primer3web version 4.1.0 (Untergasser et al., 2012), we designed primers for *PEN2*, *TGG2*, *GTR1A2*, and *GTR2A2*. A list of genes and primers is presented in **Table S3**. RNA was extracted from each of the pooled samples of ground and freeze-dried root material using innuPREP Plant RNA Kit (Analytik Jena, Jena, Germany) according to the manufacturer’s instructions. Qualitative and quantitative RNA identification was done by gel electrophoresis (1% agarose) and a NanoPhotometer^®^ P330 (Implen, Munich, Germany). We treated 5 µg of the extracted RNA with 2 U DNAse I (Thermo Fisher Scientific, Schwerte, Germany) according to the manufacturer’s instructions. We checked whether DNA was completely removed from each RNA extract with gel electrophoresis (1% agarose). cDNA was synthesized from 2 µg RNA by using 400 U Revert Aid H-Reverse transcriptase (Thermo Fisher Scientific, Schwerte, Germany) according to the manufacturer’s instructions. Samples were incubated at 42°C for 60 min, 50°C for 15 min, and finally 70°C for 15 min in a Thermal cycler (Techne, Stone, United Kingdom).

Quantitative real-time PCR (qPCR) was performed on a CFX384 Real-time system (BioRad, Munich, Germany) using a 10 µl reaction volume containing 5 µl SYBR Green qPCR master mix (2X, Thermo Fisher Scientific, Schwerte, Germany), 0.25 µl forward and reverse primer (10 µM), 0.05 µg cDNA, and 3.5 µl H_2_O. The qPCR run under following conditions: 2 min at 50°C, 5 min at 95°C and 40 cycles of 30 s at 95°C, 30 s at 58°C, and 45 s at 72°C. Thereafter, a melting curve analysis was performed to verify the amplification of a single gene transcript. Two technical replicates of each sample and no template controls were performed. All no template controls showed a Ct value larger than 39. Data were analyzed using BioRad CFX manager 3.1 software (BioRad, Munich, Germany). Expression levels were normalized to the reference gene *TIP41*.

#### GSL Analysis

To test GSL responses to root herbivore specialists, we analyzed the qualitative and quantitative composition of GSL profiles in low and high GSL varieties. GSLs were extracted from pooled samples of ground material and detected as described above. This time, we adjusted the HPLC elution gradient in order to improve the separation of the peaks for gluconasturtiin and 4-methoxyglucobrassicin. We used H_2_O as solvent A and acetonitrile as solvent B. The gradient profile started with an equilibration at 98% A for 4.3 min, followed by a gradient to 35% B within 24.3 min, hold until 29 min at 35% B. Thereafter the gradient went back to the initial 98% of A within 1 min and held the initial conditions for 10 min at 0.6 ml/min flow. The injection volume was 10 µl.

### Statistical Analyses

Analyses were performed using R version 3.2.0 (R Core Team, 2015). Data were log transformed to obtain normal distribution of the residuals. QQ- and residual plots were used to visually inspect whether residuals were normally distributed. A three-way ANOVA, with GSL varieties (low vs. high), herbivory (control, *D. radicum*, and *D. floralis*), and time point (3, 5, 7 days) as fixed factors was used to test effects on GLS biosynthesis, GSL transport, GSL hydrolysis, GSL classes, and total GSLs. Interactions of GSL varieties and treatments within time points were analyzed using a two-way ANOVA followed by a Tukey HSD post-hoc test.

## Results

### Effect of *D. radicum and D. floralis* Herbivory on GSL Biosynthesis, Transport, and Hydrolysis Gene Expression

**Figure 1:**
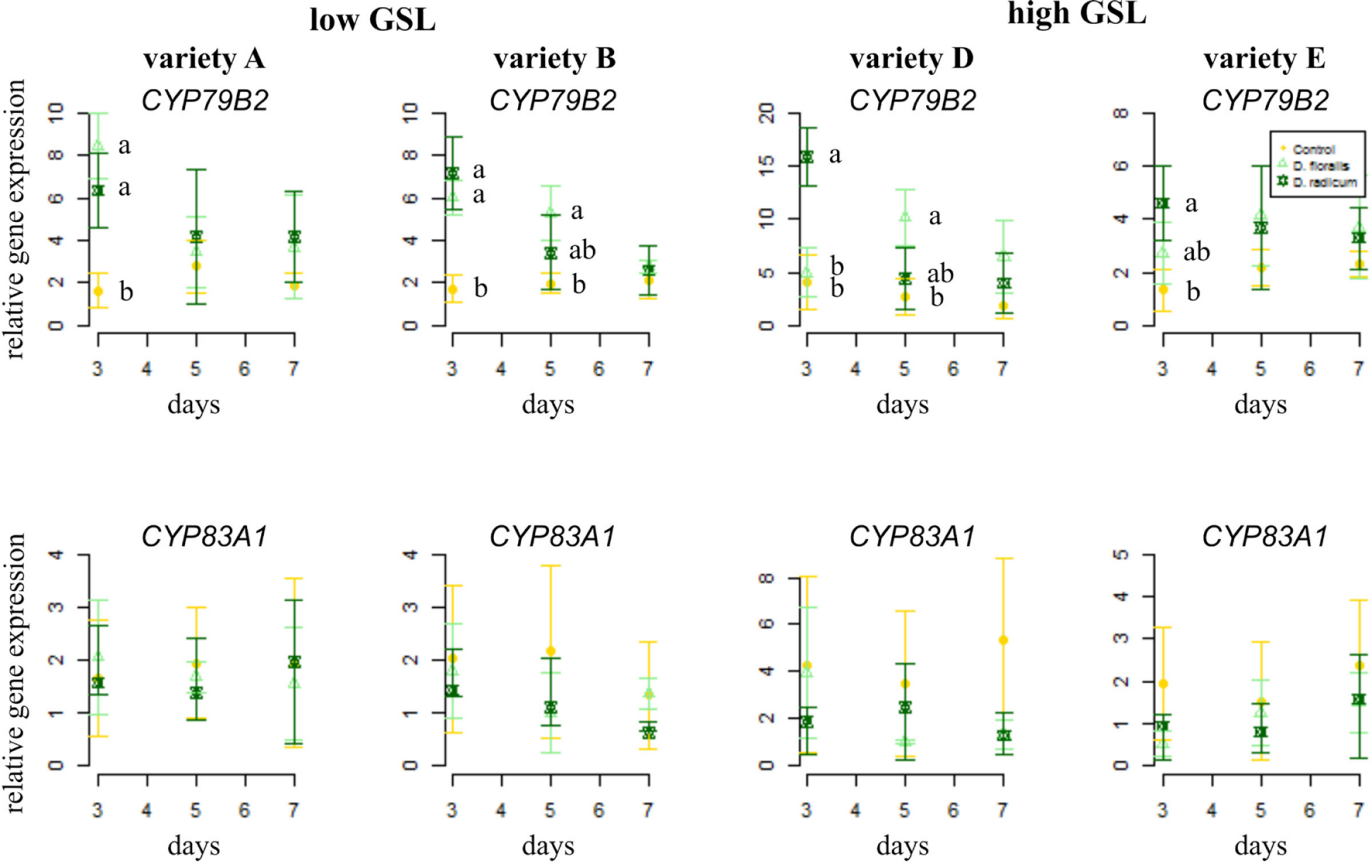
Normalized gene expression ( ± standard deviation) of the indole *CYP79B2* (top) and aliphatic *CYP83A1* (bottom) glucosinolate (GSL) biosynthesis genes in roots of low (variety **A** and **B**, left) and high (variety **D** and **E**, right) GSL varieties of *Brassica rapa*. N = 4 per variety, time point, and treatment. Different letters indicate significant differences in gene expression within that time point (p < 0.05, Tukey HSD). Roots were infested with *Delia radicum* (dark green) or *D. floralis* (light green) larvae for 3, 5, or 7 days. Control plants (yellow) were not infested. Please note the different y-axis scaling of individual graphs.

To test how low and high GSL varieties respond to *D. radicum* or *D. floralis* herbivory, we analyzed GSL biosynthesis, GSL transport, and hydrolysis gene expression in the roots of both high and low GLS varieties harvested after 3, 5, and 7 days of root herbivory. The indole biosynthesis gene *CYP79B2* was significantly upregulated in both high (variety D and E) and low (variety A and B) GSL varieties after 3 days of herbivory by *D. radicum* and reduced to control levels after 7 days in all varieties. (**Figure 1** upper row, **Table 1**, herbivory × time effect, **Table S4**). The expression of *CYP79B2* was only upregulated in low GSL varieties in response to *D. floralis* feeding after 3 days, and in variety B also after 5 days (**Figure 1**, **Table 1**, variety × herbivore × time effect). The high GSL varieties responded less strongly, and if at all later (variety D, 5 days) to *D. floralis* compared to the low GSL varieties (**Figure 1**). *D. radicum* and *D. floralis* herbivory had no significant effect on the aliphatic biosynthesis gene expression (*CYP83A1*) in both high and low GSL varieties (**Figure 1**, lower row).

**Table 1:** Test values after three-way ANOVA on glucosinolate (GSL) biosynthesis (indole *CYP79B2,* aliphatic *CYP83A1*), GSL transporter (*GTR1A2*, *GTR2A2*), and GSL hydrolysis (myrosinase *TGG2*, indole hydrolysis *PEN2*) gene expression in *B. rapa* roots of low and high constitutive GSL varieties.

Factor	Df	GSL biosynthesis genes				GSL transporter genes			
		*CYP79B2*		*CYP83A1*		*GTR1A2*		*GTR2A2*	
		F value	P value	F value	P value	F value	P value	F value	P value
GSL variety (V)	1	0.60	0.44	0.55	0.46	56.86	<0.001 ***	1.72	0.19
Herbivory (H)	2	30.19	<0.001 ***	3.46	0.03 *	7.99	<0.001 ***	2.47	0.09
Time (T)	1	8.83	0.004 **	0.08	0.77	5.42	0.02 *	3.81	0.05
V:H	2	0.08	0.93	1.41	0.25	8.08	<0.001 ***	4.37	0.01 *
V:T	1	1.44	0.23	1.29	0.26	9.10	0.003 **	0.58	0.45
H:T	2	4.97	0.008 **	0.56	0.57	8.29	<0.001 ***	6.97	0.001 **
V:H:T	2	3.42	0.04 *	0.42	0.66	0.84	0.44	7.67	<0.001 ***
		**GSL hydrolysis genes**							
		***TGG2***							
GSL variety (V)	1	13.54	<0.001 ***	0.04	0.84				
Herbivory (H)	2	8.57	<0.001 ***	2.05	0.13				
Time (T)	1	6.00	0.02 *	14.38	<0.001 ***				
V:H	2	1.96	0.14	0.98	0.38				
V:T	1	5.70	0.02 *	0.05	0.82				
H:T	2	1.99	0.14	7.99	<0.001***				
V:H:T	2	0.94	0.39	0.06	0.94				

Plants were either undamaged or induced with D. floralis or D. radicum larvae for 3, 5, or 7 days. * p < 0.05, ** p < 0.01, *** p < 0.001.

**Figure 2:**
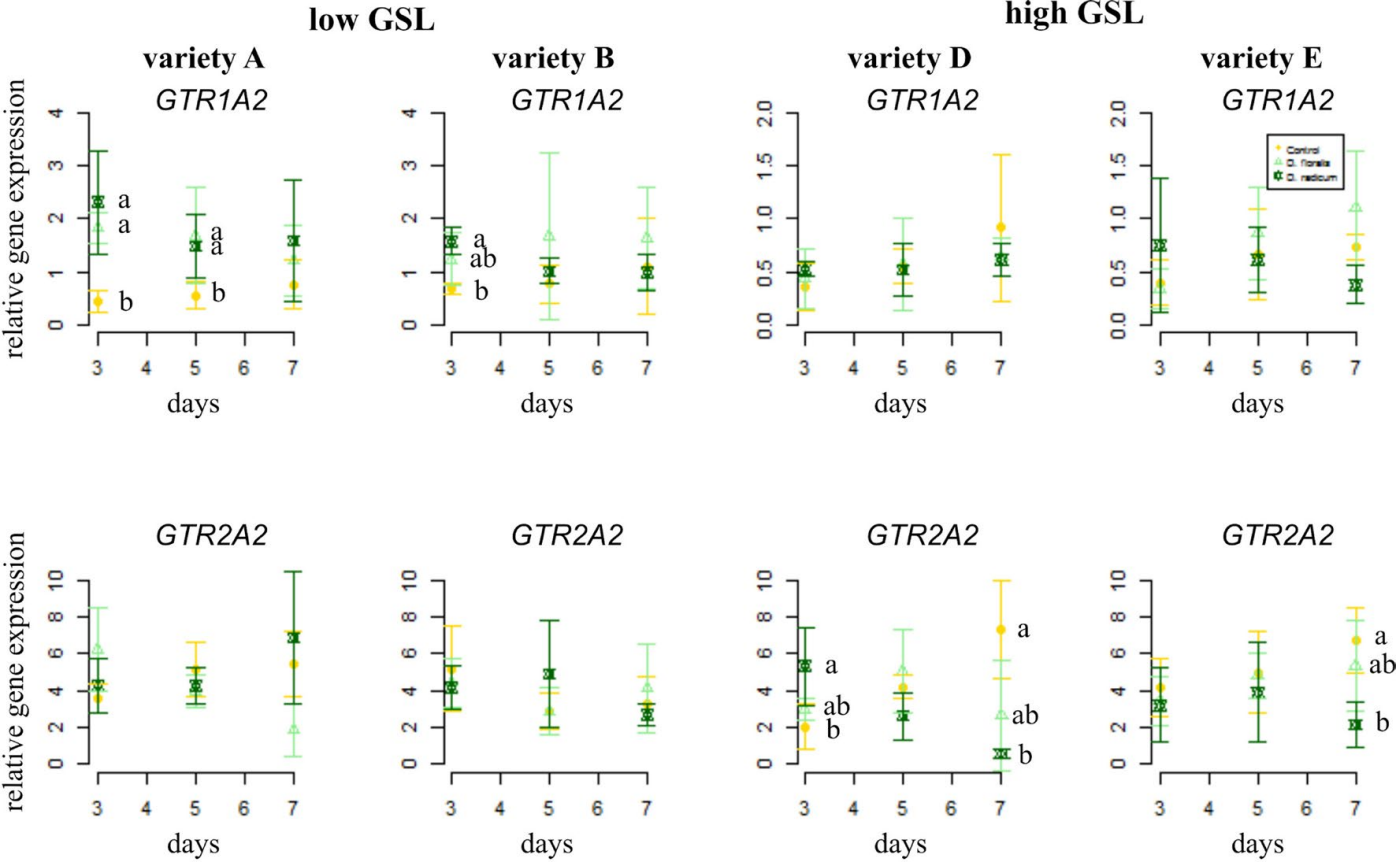
Normalized gene expression (± standard deviation) of the GSL transporters *GTR1A2* (top) and *GTR2A2* (bottom) in *B. rapa* roots of low (variety **A** and **B**, left) and high (variety **D** and **E**, right) GSL varieties. N = 4 per variety, time point, and treatment. Different letters indicate significant differences in gene expression within that time point (p < 0.05, Tukey HSD). Roots were infested with *D. radicum* (dark green) or *D. floralis* (light green) for 3, 5, or 7 days. Control plants (yellow) were not infested. Please note the different y-axis scaling of individual graphs.

In general, the GSL transporter gene *GTR1A2* was expressed at higher levels in low than in high GSL varieties (**Figure 2**, note different y-axis values, **Table 1**). Herbivory by *D. radicum* affected the expression of *GTR1A2* (**Table 1**) differently in low and high GSL varieties. Whereas *GTR1A2* expression was upregulated in low GSL varieties in response to *D. radicum* feeding after 3 days, no response was observed in high GSL varieties (**Figure 2**, **Table 1**, variety × herbivore interaction). Herbivory by *D. floralis* led to upregulated *GTR1A2* expression in the low GSL variety A after 3 days. No effects of herbivory on *GTR1A2* expression were found in high GSL varieties. The herbivore response in low GSL varieties showed temporal dynamic; the expression of *GTR1A2* was upregulated after 3 days of herbivory and dropped to control levels after 7 days in low GSL varieties (**Table 1**, herbivore × treatment interaction). The gene expression of the other transporter, *GTR2A2*, showed the opposite effect. *D. radicum* feeding only affected expression of *GTR2A2* in high GSL varieties. In variety D, *D. radicum* feeding first upregulated expression after 3 days, after which *GTR2A2* expression was downregulated after 7 days, both in high GSL varieties D and E (**Figure 2**, lower row, **Table 1**, variety × herbivore × time effect).

**Figure 3:**
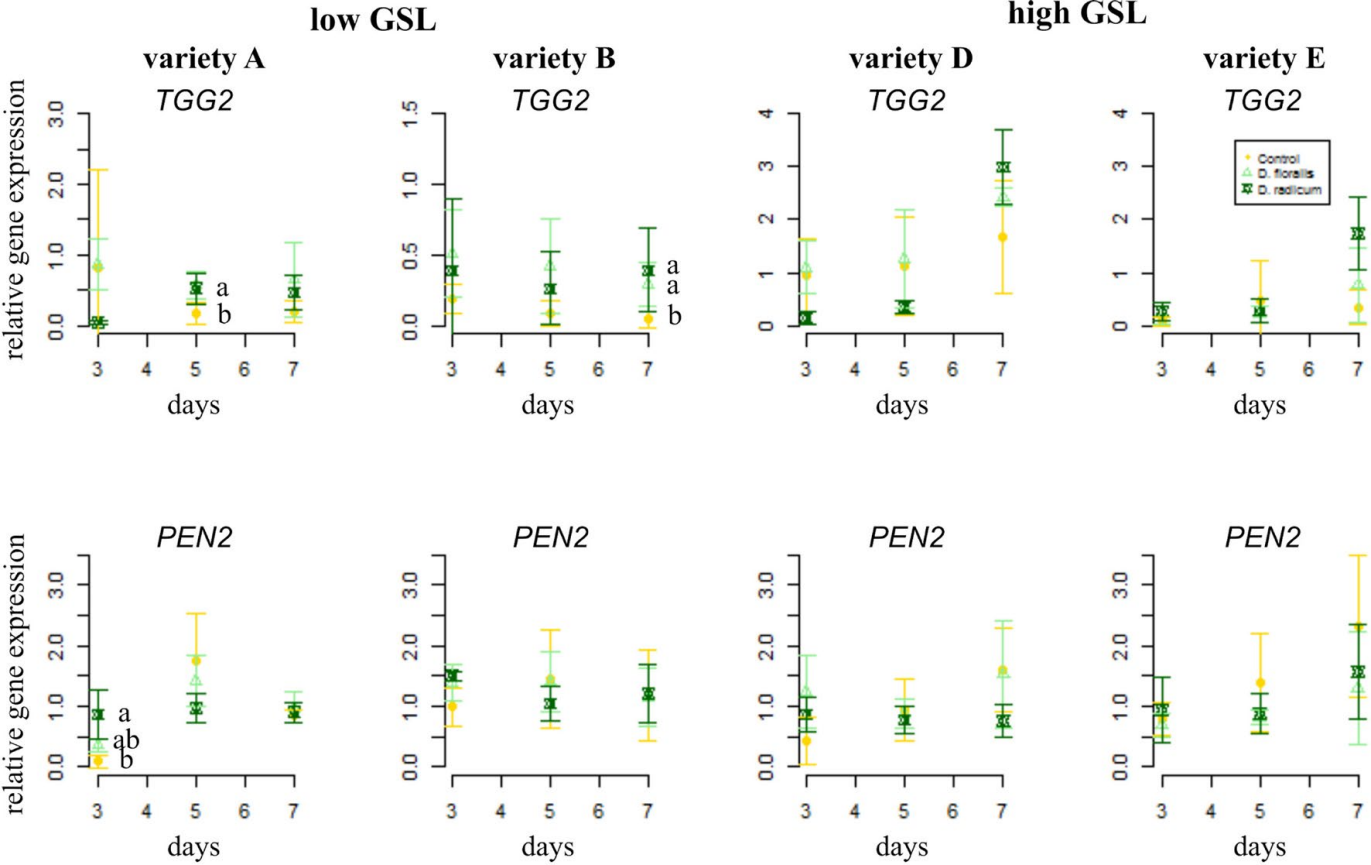
Normalized gene expression (standard deviation) of the GLS hydrolysis pathways; myrosinase gene *TGG2* (top) and indole hydrolysis gene *PEN2* (bottom) in roots of low (variety **A** and **B**, left) and high (variety **D** and **E**, right) GSL varieties of *B. rapa*. N = 4 per variety, time point, and treatment. Different letters indicate significant differences in gene expression within that time point (p < 0.05, Tukey HSD). Roots were infested with *D. radicum* (dark green) or *D. floralis* (light green) for 3, 5, or 7 days. Control plants (yellow) were not infested. Please note the different y-axis scaling of individual graphs.

The gene *TGG2*, encoding for myrosinase, showed higher base expression levels in high GSL varieties than in low GSL varieties (**Figure 3** and **Table 1**). *D. radicum* and *D. floralis* herbivory upregulated the *TGG2* expression in low GSL varieties at later time points (variety A after 5 days, variety B after 7 days). High GSL varieties did not alter *TGG2* expression upon root herbivory, through there was non-significant trend toward upregulation of *TGG2* at 7 days after *D. radicum* or *D. floralis* feeding in variety D and E (**Table 1**). *PEN 2,* a gene involved in indole GSL hydrolysis, was upregulated in response to 3 days of *D. radicum* and *D. floralis* feeding in low GSL variety A only (**Figure 3** lower row).

### Effect on GSL Levels and Profiles

**Figure 4:**
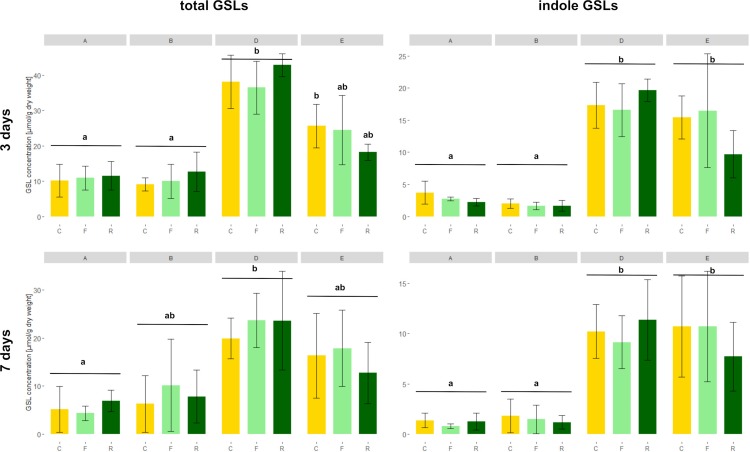
Total (left) and indole (right) GSL concentrations (micromoles g−1 dry mass ) in *B. rapa* roots of low (variety **A** and **B**) and high (variety **D** and **E**) GSL varieties. N = 4 per variety, time point, and treatment. Different letters indicate significant differences in GSL concentration within that time point (p < 0.05, Tukey HSD). Plants were infested with *D. radicum* (R, dark green) or *D. floralis* (F, light green) larvae. Control plants (C, yellow) were not infested. GSLs were measured after 3 (first column) and 7 days (second column) of herbivory. Please note the different scales on the y-axis for time points and GSL levels.

As expected, high GSL varieties had significantly higher total GSL concentrations in the roots than low GSL varieties at 3 days, except for the *D. floralis* and *D. radicum* infested plants of variety D (**Figure 4** and **Table 2**, variety effect, **Table S4**). Total GSL concentration was not significantly different at 5 and 7 days in low and high GSL varieties, except for variety A and D at 7 days (control and *D. floralis* treatment; **Figures 4** and **S2**). The high GSL concentration in the high GSL varieties were mainly due to high levels of indole GLSs in varieties D and E. Indole GSLs significantly differed in low and high GSL varieties at 3 and 7 days (**Figure 4** and **Table 2**). Aliphatic GSL levels were significantly lower in herbivore-infested roots of variety B (low GSL variety) than those of the high GSL varieties (variety D and E) at 5 days (**Figure S2** and **Table 2**). Benzyl GSLs showed a significant variety effect only at 3 days; the levels of benzyl GSLs were lowest in variety A (low GSL variety) and the highest in variety D (high GSL variety, **Figure S3**). Total, indole, and benzyl GSL concentrations fluctuated with time after harvest (**Table 2**, time effect). Herbivory by *D. radicum* or *D. floralis* larvae had no significant effect on total, indole, aliphatic, or benzyl GSL concentration when compared to their control plants (**Figures 4**, **S2** and **S3**).

**Table 2:** Test values after three-way ANOVA on total GSLs and GSL classes in *B. rapa* roots of low and high GSL varieties.

Factor	Df	Total GSLs		Indole GSLs		Aliphatic GSLs		Benzyl GSLs	
		F value	P value	F value	P value	F value	P value	F value	P value
GSL variety (V)	1	86.69	<0.001 ***	214.81	<0.001 ***	17.49	<0.001 ***	34.14	<0.001 ***
Herbivory (H)	2	0.59	0.56	0.25	0.78	4.58	0.01 *	0.39	0.68
Time (T)	1	28.71	<0.001 ***	19.83	<0.001 ***	3.98	0.05	46.55	<0.001 ***
V:H	2	1.01	0.37	0.04	0.96	0.27	0.76	1.03	0.36
V:T	1	0.40	0.53	1.13	0.29	0.27	0.60	1.28	0.26
H:T	2	0.30	0.74	0.24	0.79	0.93	0.40	0.01	0.99
V:H:T	2	0.21	0.81	0.15	0.86	0.78	0.46	0.37	0.69

Plants were either undamaged or induced with D. floralis or D. radicum larvae for 3, 5, or 7 days. * p < 0.05, ** p < 0.01, *** p < 0.001.

## Discussion

We studied induced GSL defense responses in low and high GSL containing *B. rapa* varieties upon root herbivory by *D. radicum* and *D. floralis*. Our results show that roots of high and low GSL varieties both responded to *D. radicum* feeding by upregulating *CYP79B2*, a marker gene for indole GSL biosynthesis, after 3 days. Low GSL varieties showed a faster response to *D. floralis* herbivory than high GSL varieties. Expression of *CYP83A1*, a gene involved in aliphatic GSL biosynthesis, was not induced by root fly feeding. Root fly feeding also altered the expression of two GSL transporter genes; *GTR1A2* expression was upregulated particularly in low GSL varieties after 3 days of feeding, whereas, *GTR2A2* was downregulated in high GSL varieties after 7 days. *TGG2*, the gene coding for the enzyme myrosinase, was induced by herbivory only in low GSL varieties after 5 or 7 days, whereas *PEN2* expression was only upregulated three days of *D. radicum* herbivory in variety A. In contrast to the gene expression patterns, *D. radicum* and *D. floralis* herbivory had no effect on GSL profiles in either low or high GSL varieties. Combined, our results show that biosynthesis, hydrolysis, and transporter genes are all affected by root herbivory with only minor differences in responses between high and low GSL-containing varieties.

On the gene level, we found that the indole biosynthesis gene *CYP79B2* was upregulated in response to *D. radicum* feeding in all varieties after 3 days, whereas only low GSL varieties responded to *D. floralis* feeding after 3 days. The late or lack of responses to *D. floralis* in high GSL varieties may indicate a trade-off between constitutive and induced GSL levels, although this potential trade-off seems to be herbivore-specific. In contrast to the indole biosynthesis upregulation, herbivory did not lead to significant accumulation of indole GSLs in either low or high GSL varieties. Because of the lack of indole GSLs accumulations upon *D. radicum* or *D. floralis* feeding, we found no trade-offs between constitutive and induced indole GSLs. Our findings contrast with earlier studies in *B. oleracea*, *B. nigra*, and *B. napus*, reporting that upon *D. radicum* and *D. floralis* infestations, both indole and aliphatic GSLs can increase (Birch et al., 1990; Hopkins et al., 1998; van Dam and Raaijmakers, 2006; Tsunoda et al., 2018).

Discrepancies between GSL accumulation and GSL biosynthesis gene expression are not uncommon, as similar observations were reported for *A. thaliana* shoots damaged by aboveground herbivores (Rasmann et al., 2015). Inconsistencies between gene transcription and final product may be caused by disruptions in downstream processes, including modifications in RNA processing and post-translational protein modifications (Mazzucotelli et al., 2008). A second explanation may be that we did not sample the roots on a fine enough scale, as we harvested whole root systems. This may have diluted the induced GSL signature, in particular of indole GSLs which are rather minor constituents of the total GSL profile. Previous studies showed that only taproots, and not lateral and fine roots respond to root fly feeding by increasing GSL levels (van Dam and Raaijmakers, 2006). Fine roots, on the other hand, do not respond to root fly feeding, but contain relatively high levels of indole GSLs (Tsunoda et al., 2018). By analyzing the entire root, we pooled organ-specific GSL profiles, which may have obscured local responses in the taproot. Moreover, indole GSL levels varied considerably among replicates. By pooling the two replicates from one batch, we may have reduced our statistical power. Lastly, other processes than biosynthesis, such as GSL transport and hydrolysis, may have affected local indole GSL levels. This mismatch between biosynthesis pathway activation and GSL production in our experiment shows that analyzing the biosynthesis pathway, but not the resulting GSLs may result in misleading conclusions. We therefore recommend combining both approaches to obtain ecologically relevant results.

The expression of *GTR1A2* and *GTR2A2*, both transporters with affinity to indole and aliphatic GSLs, responded differently to root fly feeding in low and high GSL varieties (Jørgensen et al., 2017). Expression of *GTR1A2* was upregulated after three days of root fly feeding in low GSL varieties, whereas expression of *GTR2A2* was downregulated in high GSL varieties after seven days. This observation supports the hypothesis that low GSL varieties show an early and strong defense response potentially to compensate for low constitutive GSL levels. Varieties with high constitutive GSL levels are already well defended and may not need additional GSL transport to the site of herbivore attack (Madsen et al., 2014). However, the direction in which GSL transporters work, or in other words whether the upregulation of *GTR* genes results in enhanced export or import of GSL to the organ in which the transcriptional changes are observed, is as yet unclear (Touw et al., submitted to same issue).

Enhanced GSL breakdown might also contribute to the observed discrepancy between the enhanced expression of GSL biosynthesis genes and GSL accumulation in herbivore-induced roots. Upon herbivory, GSLs can be hydrolyzed to more effective breakdown products such as isothiocyanates and nitriles (Agrawal and Kurashige, 2003; Bones and Rossiter, 2006). We found that expression of the myrosinase gene *TGG2* which hydrolyzes a broad range of GSLs, is only upregulated in low GSL varieties in response to root fly feeding. However, *TGG2* upregulation occurred later than the activation of GSL synthesis genes. *PEN2*, which specifically hydrolyzes indole GSLs, showed hardly any response to root fly feeding. *D. radicum* feeding enhances (iso)thiocyanate emissions from *B. rapa* roots (Crespo et al., 2012). Our findings suggest that mainly *TGG2* is involved in the hydrolysis of root GSLs in *D. radicum* and *D. floralis* infested plants.

GSL defense responses in plants can differ between different herbivore species, depending on host specialization levels (generalist, specialist), and feeding mode (sucking, chewing) (Arany et al., 2008; Bidart-Bouzat and Kliebenstein, 2011; Danner et al., 2018). In our experiment, both herbivores used (*D. radicum* and *D. floralis)* are from the same family, specialized on the same host plants, and both mine in the roots (McDonald and Sears, 1992). *D. floralis* has a larger body size and weight and thus may causes more damage than *D. radicum,* which potentially can affect plant defense responses. Our results showed that in general, *B. rapa* varieties responded similar to both *Delia* species. Species-specific responses were detected only in high GSL varieties: the expression of *CYP79B2* was upregulated earlier, whereas *GTR2A2* was downregulated mainly upon *D. radicum* feeding. This suggests that the GSL defense pathways responded partly herbivore-specific.

GSLs play an important role in plant–herbivore interactions. Negative correlations between GSL concentration and aboveground herbivore performance have been found mainly for aboveground generalists (Giamoustaris and Mithen, 1995; Arany et al., 2008). Other studies showed that aboveground generalists and specialists performed similarly on high and low GSL containing plants (Poelman et al., 2008). The production of GSLs may be costly for plants, and plants with high constitutive GSL levels may grow less well than those with low GSLs (Bekaert et al., 2012). Inducing defenses upon herbivore attack is considered a viable strategy to reduce such costs (Backmann et al., 2019). However, to invest in production upon herbivory by well-adapted specialists such as *D. radicum* or *D. floralis* larvae, which are able to detoxify GLS and their breakdown products (Welte et al., 2016), may be a waste of resources. A further reason for the lack of GSL induction in our experiment may be that GSLs and their breakdown products also act as oviposition attractants to specialists (de Vos et al., 2008; Pierre et al., 2013). Infestation by *D. radicum* increased the attractiveness of broccoli plants to ovipositing female flies (Pierre et al., 2013). We suggest that plants recognized the belowground specialist and prevented GSL accumulations that attract more and/or other above- and belowground specialists.

To conclude, low and high GSL varieties of *B. rapa* responded similarly to *D. radicum* infestation in terms of activation of indole biosynthesis genes. However, both varieties responded differently to *D. floralis* infestations in terms of GSL transporter and indole biosynthesis gene expression. Our hypothesis that there might be a trade-off between constitutive and induced GLS defenses in roots, similar to what has been found in shoots (Rasmann et al., 2015), is thus not supported by our experiments. In other words, low GSL varieties did not compensate for lower constitutive GSL concentrations with a higher accumulation of induced GSLs upon *D. radicum* or *D. floralis* infestation to attain similar GSL levels as high GSL varieties. Finally, we found that the variation in root GSL levels among *B. rapa* varieties is larger than that for shoots. Several cultivated *B. rapa* varieties which are used for human and livestock consumption are selected for low seed or leaf GSL levels. Low GSL levels in the roots are likely non-target side effects of the breeding process, which may result in varieties that are more susceptible to root herbivores and pathogens (Potter et al., 1999; Kabouw et al., 2010). Therefore it is recommended that root GSL levels as well as their induced responses to generalist and specialist root herbivores should be considered when breeding GSL containing crops.

## Data Availability Statement

All datasets generated for this study are included in the article/**Supplementary Material**.

## Author Contributions

ND and RS designed the project. RS and NG performed the experiment, extraction, and data analysis. AS developed the HPLC method. SP provided plants, designed, and tested primers. ND, AT, and RS interpreted the data. All authors contributed to the writing of the manuscript.

## Funding

Financial support by Friedrich Schiller University Jena/German Research Foundation (DFG) Collaborative Research Center 1127 ChemBioSys and DFG funding to the German Centre for Integrative Biodiversity Research (iDiv) Halle-Jena-Leipzig (FZT 118) is gratefully acknowledged. We thank the “Thüringer Universitäts-und Landesbibliothek Jena” (THULB) for their contribution to the publication fee.

## Conflict of Interest

The authors declare that the research was conducted in the absence of any commercial or financial relationships that could be construed as a potential conflict of interest.
