# Supplementary material for: Same Difference? Low and High Glucosinolate Brassica rapa Varieties Show Similar Responses Upon Feeding by Two Specialist Root Herbivores
Source: Front Plant Sci. 2019 Nov 13;10:1451. doi: 10.3389/fpls.2019.01451 (PMC6865846; doi:10.3389/fpls.2019.01451)
Supplement: Supplementary file 1 [file DataSheet_1.pdf]

Supplementary material

TABLE S1 *Brassica rapa* accessions from the seedbank IPK Gatersleben, Germany and Baaij et al. (2018) which were tested about their GSL content.

Accession number	Species	Accession name	Origin country	biostatus	ID
BRA 1771	*Brassica rapa* L.	cime di rapa	Italy	local variety	A
BRA 1772	*Brassica rapa* L.	cime di rapa	Italy	local variety	B
BRA 1773	*Brassica rapa* L.	cime di rapa	Italy	local variety	F
BRA 1780	*Brassica rapa* L.	Broccoletto o Cima di Rapa	unknown	commercial crop	G
BRA 1828	*Brassica rapa* L.	Maruba Wase Komatsuna	Unknown	commercial crop	H
BRA 1903	*Brassica rapa* L.	Komatsuma	Japan	local variety	C
BRA 1904	*Brassica rapa* L.	Narovit Greens	Unknown	commercial crop	I
BRA 2810	*Brassica rapa* L.	unknown	China	local variety	J
BRA 2838	*Brassica rapa* L.	rapa	Italy	local variety	D
-	*Brassica rapa* L.	Maarssen	Netherlands	wild variety	E

TABLE S2 Reference standards of desulfo-glucosinolates, their response factors and approximate retention times (Rts) on ThermoFisher/Dionex Ultimate HPLC platforms equipped with an C18 column (150 x 4.6 mm, 3 micrometer particle size) plus C_{18} precolumn (10 x 4.6 mm, 5 micrometer particle size). Eluents, gradient, column temperature, and flow rate as described in material and methods. Rt rounded to nearest 0.1 min (± 0.3 min depending on the column, eluent quality). Peaks, Rt and UV spectra of glucosinolates marked with # were compared with those of commercially bought references (Phytoplan, Heidelberg, Germany).

Abreviation	Common name	Side chain structure	Rt (min)	229nm	Reference
	aliphatic glucosinolates				
SIN	Sinigrin#	2-propenyl	7.060	1	Brown et al. (2003), European Community (1990)
GNA	Gluconapin#	3-butenyl	12.850	1.11	European Community (1990)
GBN	Glucobrassicanapin#	4-pentenyl	16.150	1.15	European Community (1990)
IBV	Glucoiberverin	3-methylthiopropyl	14.187	0.8	Brown et al. (2003)
ERU	Glucoerucin	4-methylthiobutyl	16.583	0.9	Brown et al. (2003)
RAPH	Glucoraphanin#	4-methylsulfinylbutyl	6.160	0.9	Brown et al. (2003)
ALY	Glucoalyssin#	5-methylsulfinylpentyl	10.937	0.9	Brown et al. (2003)
ARA	Glucoarabin#	9-methylsulfinylnonyl	20.080	1	
EPI	Epiprogoitrin#	2(S)-OH-3-butenyl	6.333	1	
PRO	Progoitrin#	2(R)-OH-3-butenyl	5.670	1.09	Buchner (1987), European

					Community (1990)
	indole glucosinolates				
4OH	4-hydroxyglucobrassicin#	4-hydroxyindol-3-ylmethyl	13.260	0.28	Buchner (1987), European Community (1990)
GBC	Glucobrassicin#	indol-3-ylmethyl	17.423	0.29	Buchner (1987), European Community (1990)
4MeOH	4-Methoxyglucobrassicin#	4-methoxyindol-3-ylmethyl	19.070	0.25	Buchner (1987), European Community (1990)
NEO	Neoglucobrassicin#	1-methoxyindol-3-ylmethyl	21.970	0.2	Buchner (1987), European Community (1990)
	benzyl glucosinolates				
NAS	Gluconasturtiin#	2-phenylethyl	19.340	0.95	Buchner (1987), European Community (1990)

TABLE S3 Primer names, sequences, amplicon length and reference for genes involved in indole and aliphatic GSL biosynthesis, GSL transporter and GSL hydrolysis.

**Symbol**	**type**	**Sequence**		**length**	**Reference/Accession number**
TIP41	housekeeping	Fw	TGCGAAAGGGTATCCAGTTG	~ 100bp	T. O. Tytgat, unpublished data/ Bra011516
		Rv	ATCACCGGAAGCCTCTGAC		
CYP79B2	Indole GSL biosynthesis	Fw	AAGAGGTTGTGCTGCTCCG	~ 100bp	Tytgat et al., 2013/ At4g39950
		Rv	TCCAAGTGAAACCTTGAAGAAGTC		
CYP83A1	aliphatic GSL biosynthesis	Fw	CTCCTTATCCCTCGTGCTTG	~100bp	Mathur et al., 2013 At4g13770
		Rv	TGTCGTAACCAGCGATCTTG		
PEN2	GSL hydrolysis (indole GSLs)	Fw	ACGGAGAATGGGTATGGTGA	117bp	This paper/ Bra004839
		Rv	TTGATGAATGGCGTGGATGT		
TGG2	GSL hydrolysis (Myrosinase)	Fw	CCAAATACGGCGACCCTTTA	147bp	This paper/ Bra020549
		Rv	TGTAGTTGATCACCTTGCGG		
GTR2A2	GSL transporter	Fw	GTACACAGGACGACGCAGAA	299bp	This paper/ Bra029248
		Rv	GTTGTAAATGGTATGAAGCTGACCA		

GTR1A2	GSL transporter	Fw	ATTCACCTTCGGGGAACTGG	201bp	This paper/
		Rv	TCGCTTGCTTCTGCTTGGTC		Bra018096

TABLE S4 Concentration of total GSLs and GSL levels in µmol g^{-1} dry weight, relative gene expression of GSL biosynthesis genes (indole: *CYP79B2*, aliphatic: *CYP83A1*), transporters (*GTR1A2*, *GTR2A2*) and hydrolysis (*PEN2*, *TGG2*) in the roots of four *B. rapa* varieties (A, B, D, E). Roots were non-infested (C), infested with D. radicum (Dra) or infested with D. floralis larvae (DFl) for 3, 5 or 7 days.

variety	treatment	time	Total GLSs	Aliphatic GSLs	Indole GSLs	Benzyl GSLs	*CYP79B2*	*CYP83A1*	*GTR1A2*	*GTR2A2*	*PEN2*	*TGG2*
A	C	3	17.95887	5.8964	5.2119	6.8506	2.796	2.793	0.411	4.253	0.001	0.003
A	C	3	7.582256	1.168	5.5659	0.8483	1.212	2.215	0.261	3.783	0.077	2.904
A	C	3	9.216333	3.1269	3.0262	3.0632	1.43	1.273	0.738	3.936	0.018	0.083
A	C	3	5.823061	3.2129	1.084	1.5261	0.988	0.287	0.35	2.469	0.233	0.275
A	DFl	3	16.67985	7.7767	2.9175	5.9856	10.12	3.356	1.806	4.453		0.579
A	DFl	3	7.978655	3.2539	2.2295	2.4953	6.339	1.708	1.4	4.431	0.392	0.627
A	DFl	3	9.956093	4.9239	2.9163	2.1159	8.512	0.765	2.002	6.792	0.216	0.898
A	DFl	3	9.14647	3.432	2.8132	2.9012	8.84	2.371	2.087	9.186	0.438	1.353
A	Dra	3	17.54764	6.829	3.1734	7.5452	7.6	1.697	1.329	3.123	0.484	0.066
A	Dra	3	12.86552	4.4751	2.1127	6.2778	8.071	3.021	1.878	3.494	1.115	0.069
A	Dra	3	7.326189	3.9205	1.624	1.7817	4.706	0.557	2.454	6.44	0.526	0.038
A	Dra	3	8.324087	4.1399	1.9824	2.2018	5.05	0.978	3.606	3.924	1.304	0.027
B	C	3	6.239449	1.5538	1.399	3.2866	2.391	3.227	0.77	3.151	1.37	0.261
B	C	3	11.23532	3.2668	1.1276	6.8409	1.924	3.224	0.534	7.47	0.783	0.256
B	C	3	8.696045	2.4623	2.6208	3.6129	1.673	0.909	0.736	6.92	0.698	0.035
B	C	3	10.21718	3.3602	2.8907	3.9663	0.836	0.715	0.671	3.166	1.089	0.211
B	DFl	3	17.99307	7.8665	1.3737	8.753	6.361	1.146	0.618	2.828	1.403	0.511
B	DFl	3	9.334474	2.8619	2.6363	3.8363	5.011	0.903	1.121	4.742	1.599	0.441
B	DFl	3	6.260763	2.2211	1.0215	3.0181	6.845	2.749	1.801	5.981	1.589	0.162
B	DFl	3	6.297741	2.904	1.5619	1.8319	5.84	2.371	1.387	4.148	0.955	0.912
B	Dra	3	17.78212	7.2265	1.9121	8.6435	9.284	2.413	1.237	4.398	1.536	0.142
B	Dra	3	18.10436	6.4615	2.8985	8.7443	6.571	1.643	1.764	5.62	1.547	0.109
B	Dra	3	9.839675	4.3914	1.4281	4.0203	5.212	0.731	1.786	3.993	1.524	0.133
B	Dra	3	4.980309	3.2012	0.4843	1.2949	7.589	0.925	1.498	2.678	1.392	1.161
D	C	3	28.41445	5.9895	13.0455	9.3794	2.306		0.562	2.564	0.002	0.129
D	C	3	44.84115	6.9933	15.3517	22.4961	7.6324	0.23	0.027	0.244	0.21	0.726
D	C	3	46.13563	4.8681	22.5598	18.7078	2.25	7.637	0.436	2.406	0.821	1.253
D	C	3	33.29479	3.9338	18.2919	11.0691	4.106	5.022	0.399	2.888	0.716	1.7213
D	DFl	3	46.90699	7.9789	23.4092	15.5188	6.065	1.536	0.245	2.272	0.586	0.791
D	DFl	3	40.18542	9.7696	14.324	16.0918	2.677	4.596	0.147	2.806	2.005	1.138
D	DFl	3	29.11754	3.2918	15.7447	10.0811	3.44	7.607	0.69	3.559	1.036	1.78
D	DFl	3	29.74564	6.7556	12.7485	10.2415	7.72	2.065	0.671	3.404	1.349	0.687
D	Dra	3	44.07755	10.6734	19.0859	14.3182	13.978	1.257	0.48	8.472	0.536	0.663
D	Dra	3	47.34723	9.4702	21.3123	16.5647		1.518	0.47	4.367	1.21	0.181
D	Dra	3	38.67347	8.1674	17.0069	13.4992	19.04	2.451	0.594	4.607	0.73	10.5
D	Dra	3	41.34493	7.788	21.1425	12.4145	14.654	2.318	0.565	3.87	0.956	0.256

variety	treatment	time	Total GLSs	Aliphatic GSLs	Indole GSLs	Benzyl GSLs	*CYP79B2*	*CYP83A1*	*GTR1A2*	*GTR2A2*	*PEN2*	*TGG2*
E	C	3	19.70942	2.8997	10.0679	6.7418	2.319	1.246	0.651	4.455	1.173	0.004
E	C	3	36.01941	4.6603	17.6633	13.6958	1.521	3.37	0.308	6.006	0.773	0.189
E	C	3	24.17071	0.9166	18.7304	4.5237	0.544	2.685	0.155	2.167	0.547	0.081
E	C	3	22.61034	1.6796	15.3259	5.6049	0.902	0.432	0.46	4.124	0.668	0.053
E	DFl	3	21.96698	2.848	12.7521	6.3669	3.948	0.514	0.326	2.948	0.492	0.527
E	DFl	3	31.10617	3.0048	19.9293	8.172	2.156	0.912	0.59	4.195	0.868	0.26
E	DFl	3	9.551965	1.6998	4.5607	3.2914	1.397	0.209	0.296	1.734	0.533	0.064
E	DFl	3	35.17561	2.5565	28.5915	4.0276	3.442	0.384	0.135	4.77	0.83	0.12
E	Dra	3	19.36472	2.0265	12.8107	4.5275	6.054	0.716	0.316	2.584	0.402	0.524
E	Dra	3	18.67535	2.5688	12.9471	3.1595	2.668	1.22	0.346	2.518	0.533	0.143
E	Dra	3	14.32688	2.6602	9.0828	2.5839	4.715	0.65	1.674	1.509	1.431	0.232
E	Dra	3	20.4733	11.9826	3.9289	4.4583	4.991	1.109	0.674	6.156	1.361	0.159
A	C	5	8.3356	1.5649	1.7921	2.7111	4.44	2.835	0.434	6.847	2.387	0.124
A	C	5	12.1509	3.0331	4.0705	5.0998	2.67	2.442	0.277	3.289	0.747	0.416
A	C	5	7.7825	2.301	2.5153	3.3615	1.387	2.038	0.585	4.962	1.604	0.078
A	C	5	10.4727	6.3531	3.7525	3.5223	2.642	0.465	0.882	5.491	2.299	0.075
A	DFl	5	16.4199	3.6786	4.7398	4.5298	2.677	2.024	0.735	3.926	1.886	0.49
A	DFl	5	21.6224	2.6024	3.7288	9.2428	5.861	1.812	1.2	2.855	1.091	0.469
A	DFl	5	19.2357	8.0236	7.7569	5.072	3.286	1.519	2.814	4.966	1.039	0.874
A	DFl	5	11.5174	2.2964	5.0863	2.8099	2.058	1.377	1.979	4.076	1.655	0.481
A	Dra	5	15.2138	3.0194	1.614	5.3794	3.667	1.101	1.12	3.221	0.828	0.825
A	Dra	5	16.4715	4.2243	4.6036	4.4083	8.347	2.897	0.998	5.504	1.159	0.468
A	Dra	5	12.7842	1.5902	5.2858	2.7923	4.185	0.511	2.337	4.01	0.716	0.523
A	Dra	5	17.2034	9.3273	8.7378	3.9222	0.535	0.985	1.469	4.31	1.18	0.293
B	C	5	25.2604	2.1235	2.078	14.5955	2.565	3.207	0.681	3.894	2.576	0.212
B	C	5	13.9095	4.0318	1.0797	9.7891	1.86	3.907	0.415	2.41	0.748	0.057
B	C	5	6.6649	1.6422	2.7882	2.785	1.408	0.804	0.716	3.499	0.995	0.005
B	C	5	6.122	2.272	1.9457	2.2374	2.038	0.751	1.263	1.798	1.495	0.084
B	DFl	5	9.741	3.039	1.1338	4.2683	6.684	0.907	0.874	3.014	0.987	0.268
B	DFl	5	9.4581	1.4708	4.7675	2.2845	3.728	0.451	0.676	1.481	0.957	0.813
B	DFl	5	7.4647	0.6432	1.7914	3.4385	5.861	2.086	1.107	4.043	1.938	0.549
B	DFl	5	4.8275	0.6811	0.6913	1.4993	4.949	0.515	4.005		1.732	0.048
B	Dra	5	8.7504	1.0321	3.2394	3.8905	4.097	1.629	0.999	1.471	0.928	0.269
B	Dra	5	18.3262	2.8168	4.8057	6.1219	5.127	2.134	0.776	3.794	0.956	0.622
B	Dra	5	7.0195	3.4036	0.8839	2.8219	0.995	0.408	1.248	6.028	0.82	0.078
B	Dra	5	6.6742	0.6596	2.3759	1.0916	3.454	0.228	6.283	8.302	1.46	0.078
D	C	5	10.278	2.016	2.1892	3.7699	2.619	3.453	0.496	4.197	1.638	0.094
D	C	5	16.0765	4.9996	7.071	5.5785	1.126	1.232	0.785	4.071	0.95	5.274
D	C	5	18.4257	8.8509	6.6939	8.7863	1.884	7.838	0.45	3.566	0.663	1.422
D	C	5	7.7546	6.7303	3.1641	3.0284	5.013	1.32	0.457	5.004	0.47	1.875
D	DFl	5	16.0819	4.6202	3.4639	4.7735	11.446	1.082	1.224	8.396	1.113	1.807
D	DFl	5	19.9816	6.978	5.8411	6.18	9.682	0.961	0.371	4.098	0.908	0.41
D	DFl	5	19.334	8.6385	6.3555	5.8591	6.666	1.024	0.423	4.632	0.974	0.556
D	DFl	5	17.4917	6.8944	3.0519	6.1152	12.947	0.986	0.252	3.197	0.529	2.28
D	Dra	5	20.0967	6.2389	5.372	6.4668	3.495	5.05	0.284	4.439	1.031	0.506
D	Dra	5	27.4579	10.0077	7.8213	9.2963	8.015	2.301	0.43	2.417	0.598	0.264
D	Dra	5	15.6034	8.2645	4.222	6.47	5.209	1.962	0.505	2.151	0.569	0.238
D	Dra	5	14.9433	7.6793	5.259	6.2597	1.084	0.648	0.863	1.48	0.868	0.419

variety	treatment	time	Total GLSs	Aliphatic GSLs	Indole GSLs	Benzyl GSLs	*CYP79B2*	*CYP83A1*	*GTR1A2*	*GTR2A2*	*PEN2*	*TGG2*
E	C	5	18.5074	8.2306	7.5769	7.1733		1.118	0.391	2.875	0.684	1.624
E	C	5	24.0892	13.3542	15.6236	6.953	1.397	3.519	0.21	3.3	0.811	0.179
E	C	5	8.7006	3.5717	3.7757	3.8581	2.31	1.126	0.992	7.217	2.426	0.006
E	C	5	19.7247	12.1819	13.3995	4.4386	2.749	0.313	1.071	6.613	1.642	0.005
E	DFl	5	15.6939	8.9705	9.874	3.6505	6.701	1.373	0.302	3.223	0.932	0.217
E	DFl	5	7.9511	4.928	5.0109	1.4484	4.008	0.952	1.116	4.904	0.889	0.373
E	DFl	5	9.1534	4.7117	6.1446	1.8493	3.718	2.243	1.3	6.131	0.666	0.228
E	DFl	5	12.8491	7.8746	8.4537	2.4463	2.187	0.442	0.721	4.931	0.766	0.017
E	Dra	5	29.1979	14.3098	16.5991	6.2342	6.994	1.73	0.633	2.239	0.982	0.292
E	Dra	5	13.1083	7.4076	7.2861	3.6538	3.691	0.344	0.21	1.026	0.648	0.122
E	Dra	5	6.1482	2.793	3.8363	1.2468	2.128	0.303	0.65	6.895	1.312	0.119
E	Dra	5	11.861	6.7886	7.2372	2.6482	1.93	0.835	0.961	5.355	0.565	0.597
A	C	7	13.3243	5.4205	2.5822	5.321606	1.755	1.77	0.36	4.885	0.71	0.135
A	C	7	1.595	0.3913	0.8271	0.3766	2.694	4.248	0.636	6.734	0.877	0.418
A	C	7	2.7832	1.0424	1.0143	0.7265	1.653	0.982	1.413	6.968	0.819	0.051
A	C	7	2.6914	1.4625	0.935	0.294	1.321	0.751	0.605	3.239	0.926	0.211
A	DFl	7	5.8597	2.766	1.1508	1.9429	1.838	0.849	0.771	1.537	0.61	0.461
A	DFl	7	1.9142	1.101	0.5693	0.2439	2.982	1.99	0.767	2.993	1.116	1.422
A	DFl	7	5.1592	3.3945	0.5941	1.1706	7.299	2.85	2.176	0.016	1.005	0.417
A	DFl	7	4.3344	2.867	0.8602	0.6073	2.605	0.539	1.113	2.95	1.17	0.301
A	Dra	7	8.4946	5.045	0.7841	2.6655	2.835	1.411	0.547	3.145	0.743	0.141
A	Dra	7	4.5063	3.2029	0.4334	0.87	3.021	0.552	1.433	6.288	1.136	0.714
A	Dra	7	9.6755	5.3617	2.6283	1.6855	7.32	3.074	3.226	11.818	0.851	0.49
A	Dra	7	4.8543	2.8653	1.0658	0.9232	3.529	2.789	1.139	6.287	0.87	0.539
B	C	7	16.468	5.9652	4.4654	6.03732	2.883	1.24	0.686	4.923	2.125	0.027
B	C	7	3.9572	1.1412	1.9491	0.8668	1.802	1.416	0.603	4.197	0.5	0.134
B	C	7	2.0584	0.9113	0.3904	0.7568	2.854	2.587	2.474	2.051	0.676	0.011
B	C	7	2.7386	1.949	0.358	0.4316	1.02	0.121	0.645	1.743	1.394	0.01
B	DFl	7	26.3921	9.0345	3.7861		2.439	1.339	1.113	3.097	1.749	0.145
B	DFl	7	8.0604	4.8282	1.5007	1.7316	2.563	0.967	0.948	4.272	0.776	0.283
B	DFl	7	3.0398	1.6664	0.3258	1.0476	2.922	1.593	1.433	1.637	0.743	0.506
B	DFl	7	3.0873	2.6024	0.1978	0.2871	3.09	1.561	3.045	7.4	1.283	0.238
B	Dra	7	17.2173	10.532	2.3086	4.3767	3.708	0.417	0.837	2.577	1.776	0.302
B	Dra	7	5.9404	3.5686	0.9794	1.3923	3.31	0.865	0.589	2.163	0.612	0.829
B	Dra	7	4.9111	3.0047	0.789	1.1174	1.129	0.598	1.272	3.533	1.367	0.198
B	Dra	7	3.1068	1.9966	0.5481	0.5621	2.27	0.642	1.24	2.516	1.08	0.235
D	C	7	17.8587	2.9313	8.6638	6.2636	1.359	2.645	0.566	5.403	2.368	1.716
D	C	7	27.1438	3.0418	14.7401	9.3619	1.381	7.348	0.582	4.693	1.773	7.01
D	C	7	18.4779	4.324	9.6596	4.4942	3.601	2.271	1.96	9.914	0.693	0.579
D	C	7	16.1682	1.9128	7.8498	6.4056	1.152	9.245	0.551	9.387	1.584	2.698
D	DFl	7	27.7248	10.3512	9.158	8.2157	8.38	0.803	0.615	2.957	2.835	2.575
D	DFl	7	23.6647	9.0526	9.9148	4.6973	10.074	2.005	0.601	6.767	0.94	2.184
D	DFl	7	14.5005	6.5147	5.1149	2.8709	4.574		0.462	0.505	1.181	2.486
D	DFl	7	28.8947	10.1172	12.4335	6.344	2.733	1.238	0.881	0.399	1.119	2.426
D	Dra	7	30.1378	9.2426	13.4191	7.4762	7.214	2.513	0.47	0.838	0.894	3.805
D	Dra	7	9.2411	2.88	5.1843	1.1767	1.114	0.186	0.82	0.17	0.45	3.219
D	Dra	7	19.0569	4.7773	10.8733	3.4063	5.361	1.406	0.59	0.59	1.06	2.859
D	Dra	7	36.041	11.3513	16.0457	8.644	2.354	1.091	0.565	0.609	0.612	2.107

variety	treatment	time	Total GLSs	Aliphatic GSLs	Indole GSLs	Benzyl GSLs	*CYP79B2*	*CYP83A1*	*GTR1A2*	*GTR2A2*	*PEN2*	*TGG2*
E	C	7	31.5225	5.2474	19.319	6.9562	2.47	4.65	0.587	5.507	3.305	0.808
E	C	7	12.5395	2.1785	7.2159	3.1451	2.705	1.672	0.806	5.602	3.349	0.221
E	C	7	11.8396	0.5935	9.4579	1.7882	1.653	1.86	0.793	6.602	1.105	0.277
E	C	7	9.491	1.5348	6.8911	1.0652	2.413	1.233	4.145	9.240	1.552	0.094
E	DFl	7	28.5086	2.688	18.7557	7.0649	1.633	1.553	1.084	3.116	0.486	0.982
E	DFl	7	22.0104	4.03	12.8452	5.1352	5.973	1.171	0.662	7.724	0.913	0.273
E	DFl	7	12.8345	4.8477	6.0629	1.9239	4.669	0.794	1.876	3.393	2.652	1.655
E	DFl	7	8.0845	1.7398	5.1865	1.1582	2.552	2.45	0.788	7.291	1.142	0.145
E	Dra	7	17.9886	1.9404	11.9405	4.1076	2.571	1.305	0.577	2.637	2.463	1.034
E	Dra	7	20.0165	6.0622	9.5948	4.3594	2.82	2.008	0.203	3.161	1.195	1.757
E	Dra	7	4.8972	1.4598	2.8059	0.6314	2.763	0.213	0.243	0.401	1.061	2.414
E	Dra	7	8.0321	0.7412	6.5003	0.7906	5.031	2.742	0.474	2.449		

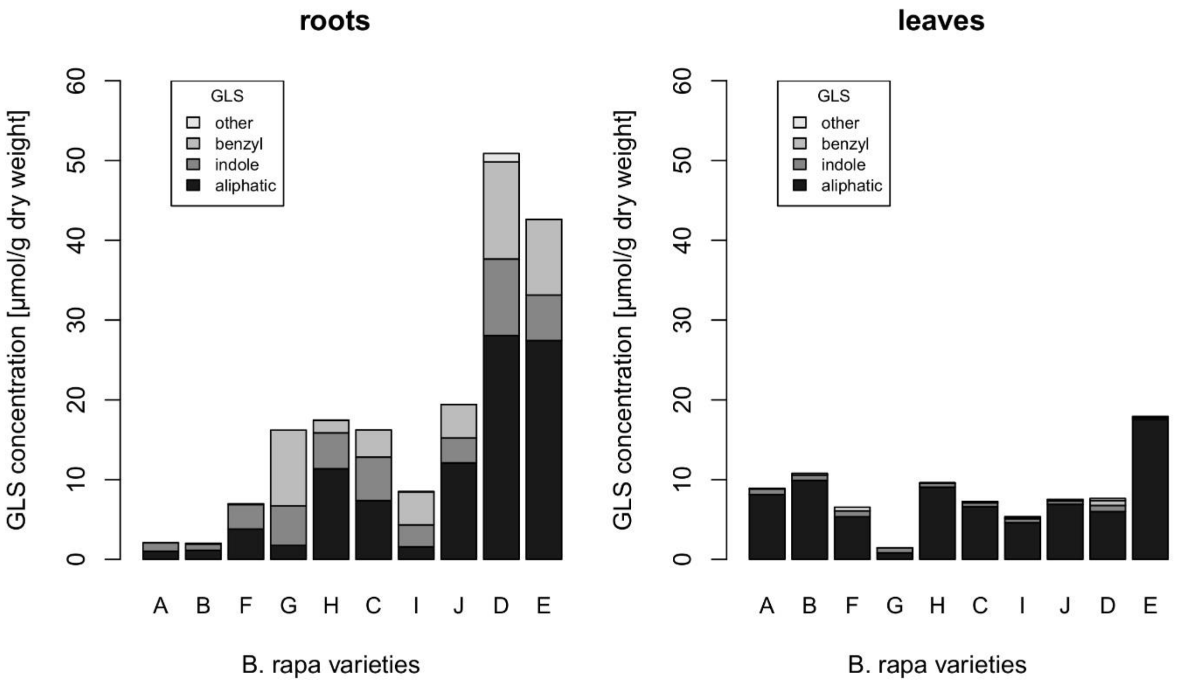

FIGURE S1 Aliphatic, indole and benzyl glucosinolate (GSL) concentration in roots and leaves of 10 *B. rapa* varieties (A-J) from the preliminary experiment.

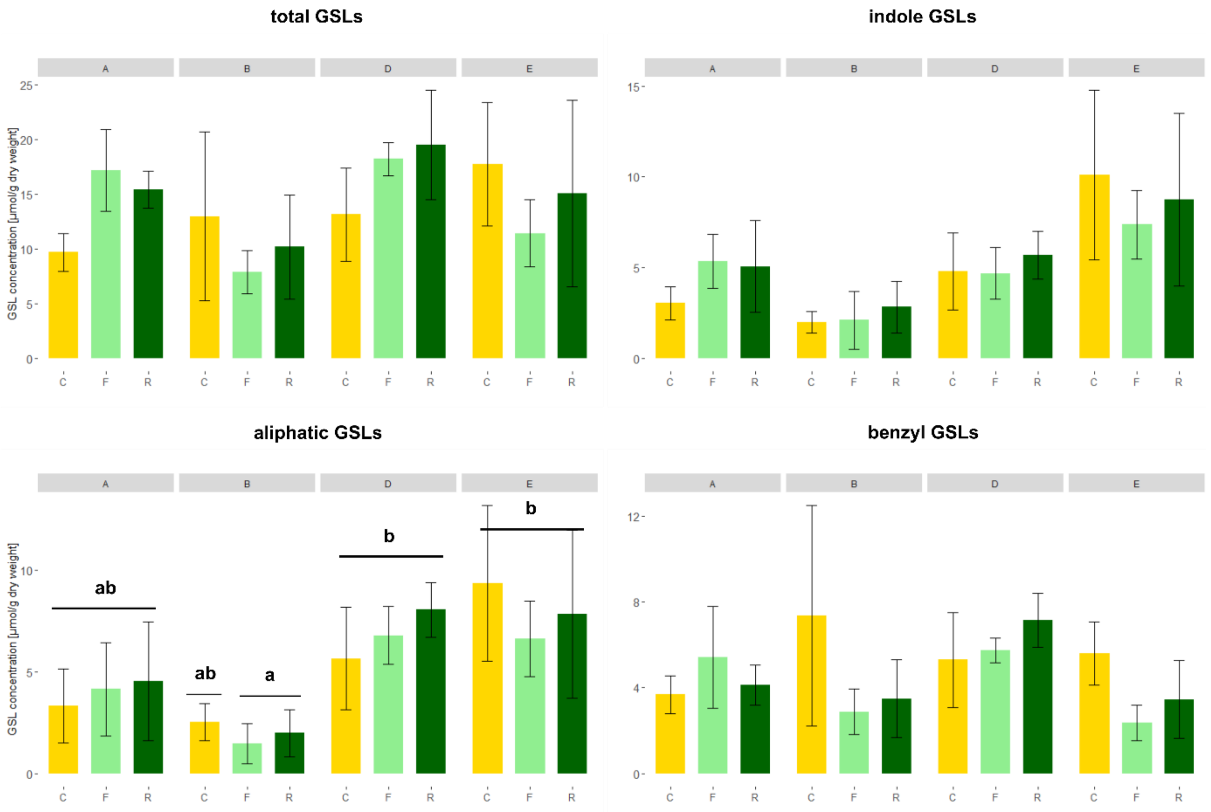

FIGURE S2 Total (first colum left), indole (first colum right), aliphatic (second column left), and benzyl (second colum right) glucosinolate (GSL) concentrations (micromoles g^{-1} dry mass) in *B. rapa* roots of low (variety A and B) and high (variety D and E) GSL accessions. Plants were infested with *Delia radicum* (R, dark green) or *D. floralis* (F, light green) larvae. Control plants (C, yellow) were not infested. GSLs were measured after 5 days of herbivory. Each treatment contained four replicates per accession and time point. Different letters indicate significant differences in GSL concentration within this time point ($p < 0.05$, Tukey HSD). Please note the different scales on the y-axis for GSL levels.

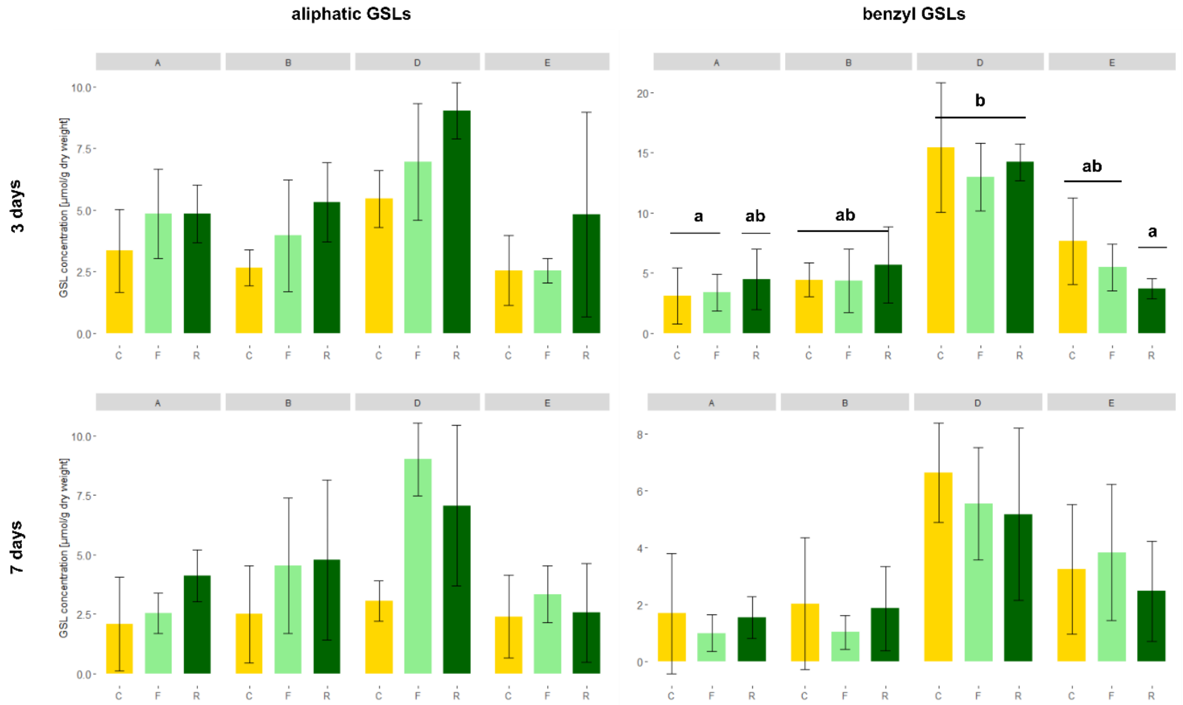

FIGURE S3 Aliphatic (left) and benzyl (right) glucosinolate (GSL) concentrations (micromoles.g^{-1} dry mass) in *B. rapa* roots of low (variety A and B) and high (variety D and E) GSL accessions. Plants were infested with *Delia radicum* (R, dark green) or *D. floralis* (F, light green) larvae. Control plants (C, yellow) were not infested. GSLs were measured after 3 (first column) and 7 days (second column) of herbivory. Each treatment contained four replicates per accession and time point. Different letters indicate significant differences in GSL concentration within this time point and GSL level ($p < 0.05$, Tukey HSD). Please note the different scales on the y-axis for time points and GSL levels.
